# Continuous light increases growth, daily carbon gain, antioxidants, and alters carbohydrate metabolism in a cultivated and a wild tomato species

**DOI:** 10.3389/fpls.2015.00522

**Published:** 2015-07-13

**Authors:** Mohammad S. Haque, Katrine H. Kjaer, Eva Rosenqvist, Carl-Otto Ottosen

**Affiliations:** ^1^Department of Food Science, Aarhus UniversityAarslev, Denmark; ^2^Department of Plant and Environmental Sciences, University of CopenhagenTaastrup, Denmark

**Keywords:** leaf chlorosis, chlorophyll fluorescence, photosynthesis, carbohydrate metabolism, antioxidative enzymes, dry mass

## Abstract

Cultivated tomato species develop leaf injury while grown in continuous light (CL). Growth, photosynthesis, carbohydrate metabolism and antioxidative enzyme activities of a cultivated (*Solanum lycopersicum* L. ‘Aromata’) and a wild tomato species (*Solanum pimpinellifolium* L.) were compared in this study aiming to analyze the species-specific differences and thermoperiod effects in responses to CL. The species were subjected to three photoperiodic treatments for 12 days in climate chambers: 16-h photoperiod with a light/dark temperature of 26/16°C (P16D10 or control); CL with a constant temperature of 23°C (P24D0); CL with a variable temperature of 26/16°C (P24D10). The results showed that both species grown in CL had higher dry matter production due to the continuous photosynthesis and a subsequent increase in carbon gain. In *S. lycopersicum*, the rate of photosynthesis and the maximum photochemical efficiency of photosystem II declined in CL with the development of leaf chlorosis, reduction in the leaf chlorophyll content and a higher activity of antioxidative enzymes. The normal diurnal patterns of starch and sugar were only present under control conditions. The results demonstrated that CL conditions mainly affected the photosynthetic apparatus of a cultivated species (*S. lycopersicum*), and to a less degree to the wild species (*S. pimpinellifolium*). The negative effects of the CL could be alleviated by diurnal temperature variations, but the physiological mechanisms behind these are less clear. The results also show that the genetic potential for reducing the negative effects of CL does exist in the tomato germplasm.

## Introduction

Providing supplemental light as continuous light (CL) in protected plant production systems can increase plant growth and development. Plants of some species exposed to CL at low photosynthetic photon flux densities (PPFDs) accumulate more dry matter than plants exposed to a shorter photoperiod at high PPFD at the same daily light integral (DLI; Oda et al., 1989; Kitaya et al., 1998; Ohyama et al., 2005a). When exposed to CL, species like tomato, potato and eggplant develop inter-vascular chlorosis and down-regulate photosynthesis (Hilman, 1956; Cao and Tibbitts, 1991; Murage et al., 1996). Furthermore, the leaf chlorophyll content was negatively correlated to the length of the photoperiod in tomato and pepper plants (Dorais et al., 1996). The maximum quantum efficiency of photosystem II (PSII) and rate of photosynthesis declined in cultivated tomato species (*Solanum lycopersicum*) while grown under CL with constant temperature (Matsuda et al., 2014; Velez-Ramirez et al., 2014).

Plants grown in CL accumulate high amounts of leaf starch and soluble sugars (Dorais et al., 1996; Globig et al., 1997; Gestel et al., 2005) and a relationship between leaf chlorosis development and starch accumulation has been suggested in tomato and eggplant (Bradley and Janes, 1985; Murage et al., 1996; Demers and Gosselin, 2002), whereas no relationship was found between these parameters in cucumber (Wolf and Langerud, 2006). Starch and sugars are diurnally regulated in plants and the timing of sampling can influence the results. In many studies on CL-grown plants the light level in the different photoperiodic treatments are identical (Bradley and Janes, 1985; Dorais et al., 1996; Demers and Gosselin, 2002), leading to differences in DLI between the treatments. An increase in light intensity may increase carbohydrate accumulation, making it difficult to separate the effect of CL and DLI. The CL-induced increase in leaf starch and concentration of hexoses have also been related to a reduced rate of photosynthesis (P_N_) and sucrose phosphate synthase (SPS) activity in tomato (Bradley and Janes, 1985; Dorais et al., 1996; Demers et al., 1998; Demers and Gosselin, 2002). The increase in leaf starch was suggested to be caused by reduced carbohydrate export from the source leaves to sinks. This increased source activity could initiate down-regulation of P_N_ at the protein or transcriptional level, thereby lowering the amount and/or activity of Rubisco (Stutte et al., 1996; Globig et al., 1997).

Since P_N_ in CL results in a continuous supply of assimilates, this could result in a hyper-accumulation of carbohydrates leading to an over-reduction of electron acceptors and a process where the photosynthetic electron transport chain donate electrons to O_2_ generating reactive oxygen species (ROS) and in turn cause oxidative damage (Cakmak and Kirkby, 2008; Velez-Ramirez et al., 2011). Tobacco plants exhibited higher ascorbate peroxidase (APX) and glutathione reductase (GR) activities when grown in CL in comparison to 12-h photoperiod conditions (Peter et al., 2010) and the catalase (CAT) enzyme activity increased faster and became significantly higher in pepper than in eggplants grown for 6 days in CL than in a control treatment (Murage and Masuda, 1997). This suggests that CL induces a higher activity of antioxidative enzymes, possibly due to a higher production of ROS. However, it has not been clarified whether the leaf chlorosis development under CL is directly related to photooxidative damage or induced by other physiological processes (Sysoeva et al., 2010).

Diurnal variations in air temperature prevented leaf chlorosis in eggplant (Murage et al., 1996), potato (Tibbitts et al., 1990; Cushman and Tibbitts, 1991; Cushman et al., 1995) and tomato (Hilman, 1956; Demers and Gosselin, 2002; Ikkonen et al., 2015). The accumulation of starch and hexoses was significantly reduced in eggplant leaves grown in CL with variations in temperature conditions of 28/15°C during 12/12-h compared to a constant temperature of 25°C, indicating that 15°C for 12-h might compensate for the dark period and restore normal physiological responses and plant growth (Murage et al., 1997). They suggested that the metabolism of starch and sugars and translocation from the leaves increase at 28/15°C. However, the role of temperature fluctuations especially the role of low temperature in preventing leaf chlorosis remains unclear even though Ikkonen et al. (2015) showed that a short term temperature drop could reset the circadian rhythms. The effect of diurnal temperature variation on diverse physiological processes in tomato under CL has not been extensively studied. In this study, we hypothesized that P_N_ would stay constant during the 24-h light period due to the constant light resulting an increase in total carbon gain and dry mass, but that the overall P_N_ rate would decline over time due to reduction in chlorophyll content. We also hypothesized that diurnal temperature variation under CL would alleviate the negative effects including leaf chlorosis development, reduction in leaf chlorophyll content and P_N_ and photooxidatative damage. The objective of this study was to investigate the effect of CL with or without diurnal temperature differences on P_N_, daily carbon gain, carbohydrate metabolism, antioxidative enzyme activities and growth (dry mass) in two tomato species during 12 days of exposure. The focus was on how CL would affect the diurnal patterns of P_N_ and leaf carbohydrate metabolism and which physiological processes would influence by temperature fluctuation under CL.

## Materials and Methods

### Plant Materials and Growth Conditions

One cultivated tomato species, *Solanum lycopersicum* L. cv. Aromata and one wild species, *Solanum pimpinellifolium* L. (unknown accession) were used in this study and the seeds of both species were collected from a web based company^1^. 150 seeds from each species were sown in June 2012 in 12 cm diameter (0.5 L) plastic pots with a commercial peat potting mix (Pindstrup 2, Pindstrup Mosebrug A/S, Ryomgaard, Denmark) under greenhouse conditions. One seed was sown per pot and the plants were kept under greenhouse conditions with a 16-h photoperiod in a combination of natural and supplemental light. Temperature set points were 26/16°C day/night and the CO_2_ level was maintained at ambient using fresh air and the relative humidity (RH) was 50–60%. The supplemental light used during seed germination and seedling establishment was ∼150 μmol m^-2^ s^-1^ provided by SON-T/400W lamp (Phillips, Amsterdam, The Netherlands). Two weeks after germination, the plantlets were moved to climate chambers (60 m^3^, Schneider Electric, 2750 Ballerup, Denmark) and kept for 1 week of acclimation in a new pattern of photoperiodic conditions with a 16-h light period maintained from 21:01 to 13:00 Central European Standard Time (CEST) and a 8-h dark period maintained from 13:01 to 21:00 CEST. The adjustment in photoperiodic conditions was done to ease of data recording time and with the assumption that diurnal patterns in plant metabolites adapt rapidly to changes in photoperiodic conditions (Kjaer et al., 2014). The light was provided by cool white fluorescent lamps (TDC, 36 W/840, Philips, Amsterdam, The Netherlands) and the temperature set points were 26/16°C, but the RH was not fully controlled. Both temperature and RH was logged during the experiment using an HMW50 sensor (Schneider Electric, Ballerup 2750, Denmark) and stored in the controller (Schneider Electric, Ballerup 2750, Denmark). Light intensity was measured manually at the canopy level with a light meter (LI-250A Quantum sensor, LI-COR, Lincoln, NE, USA) in nine different positions of the bench. The supplemental light at the canopy level was ∼225 μmol m^-2^ s^-1^.

After 3 weeks, 40 plants of each species were randomly distributed in three climate chambers (Schneider Electric, Ballerup 2750, Denmark). The plants were subjected to three treatments with different photoperiodic conditions for 12 days; (i) 16-h photoperiod with a light/dark (26/16°C) temperature difference of 10°C (DIF; P16D10 or control), (ii) CL with a constant temperature of 23°C (P24D0) and (iii) CL with a variable temperature of 10°C (P24D10). The temperature difference of 10°C in the two treatments (P16D10 and P24D10) was reached within an hour (12:30 to 13:30 and 20:30 to 21:30). Average values for temperature, RH and PPFD in the three treatments are presented in **Table 1**. The light/dark period corresponding to the 16-h light and 8-h dark in the P16D10 treatment will be referred to as time periods in all three treatments. The PPFD and temperature in the three treatments were set to maintain the same DLI (∼13 mol m^-2^ d^-1^) and the same daily mean temperature (∼23°C). An ambient level of CO_2_ using fresh air was maintained for all the treatments. The plants were watered automatically two times daily to excess with a complete nutrient solution.

**Table 1:** Daily mean (diurnal) climate data applied for the three growth conditions (P16D10, P24D0, P24D10).

Treatments	P16D10		P24D0		P24D10	
Time of day	2101-1300	1301-2100	2101-1300	1301-2100	2101-1300	1301-2100
**Light/dark (L/D)**	**L**	**D**	**L**	**L**	**L**	**L**

PPFD (μmol m^-2^ s^-1^)	223 ± 14	0 ± 0	147 ± 15	147 ± 15	148 ± 16	148 ± 16
Temperature (°C)	26 ± 2	16 ± 1	23 ± 0.1	23 ± 0.1	26 ± 2	16 ± 0.5
RH (%)	55 ± 5	51 ± 2	76 ± 5	75 ± 4	61 ± 4	56 ± 6
VPD (kPa)	1.51	0.89	0.67	0.70	1.31	0.80

*Vapour pressure deficit (VPD) is calculated on the basis of given temperature and RH. Values are mean ± SD.*

### Physiological Parameters

All physiological measurements were done on the second or third leaflets of the fourth and fifth developing leaves from three plants of each species from each of the three treatments. Leaf four and five were selected for measurement as they were expanding during the 12 days of the three different photoperiodic treatments. The selected leaflets were initially tagged. Chlorophyll fluorescence was measured using a MINI-PAM (Walz, Effeltrich, Germany) following the method described by Baker and Rosenqvist (2004). The maximum photochemical efficiency of photosystem II (PSII; F_v_/F_m_ = (F_m_-F_o_)/F_m_) was measured at the beginning of day (21:30 CEST) on day 2, 4, 8, and 12. The leaflets were dark adapted for 30 min with a leaf clip (DLC-8, Walz, Effeltrich, Germany) and F_v_/F_m_ was measured. The gas exchange measurements were done using a portable open system infrared gas analyser (CIRAS-2, PP systems, Amesbury, MA, USA). The P_N_ was measured twice a day (9:00 and 15:00 CEST) with 3 days interval using three gas-exchange systems, one placed in each climate chamber. The leaf cuvette (leaf area of 2.5 cm^2^) of the gas analyser was placed inside the climate chamber to minimize climate effects and the P_N_ was measured in the ambient PPFD and temperature conditions of each treatment. The other settings for the gas analyser were as follows: CO_2_ concentration 390 μmol mol^-1^, cuvette air flow rate 200 cm^3^ min^-1^, H_2_O content 60–80 mb. The P_N_ and g_s_ were recorded, when they reached steady state typically within 10 min.

### Leaf Pigments and Carbohydrates

The leaf samples for chlorophyll (Chl) analysis were collected at the end of the light period on day 5 and day 12 during the experiment, while the samples for carbohydrate analysis were collected twice a day (end of light and end of dark period on day 5 and day 12). The end of light (EL) refers to the end of the light period for P16D10 (12:30); the same time for P24D0 (12:30) and at the end of 26°C period for P24D10 (12:30). The end of dark (ED) refers to the end of the dark period for P16D10 (20:30); the same time for P24D0 (20:30) and at the end of 16°C period for P24D10 (20:30). The leaf samples were taken from four plants of each species in each treatment. Two small circular leaf disks (each of 2.5 cm^2^): one from base and another from the outer part of the leaflet were collected using a borer and kept in a single Eppendorf vial and used as a single sample. The collected samples were immediately plunged into liquid nitrogen and stored at -80°C until analysis. Before extraction the leaf samples were freeze dried, weighed and ground in a mixer mill (MM200, Retsch Inc., Haan, Germany) with a stainless steel ball (diameter 7 mm). The soluble sugars and pigments were extracted with 80% ethanol and 5 mM HEPES solution until the leaf material went pale. The leaf Chl content were analyzed by light spectroscopy using a UV-VIS spectrophotometer (Shimadzu, Kyoto, Japan). The Chl *a* and Chl *b* concentrations were calculated from the measurements at three absorbance levels: 470, 648.6, and 664.2 nm (Lichtenthaler, 1987). The extraction was analyzed for soluble sugars by ion chromatography using a pulsed amperometic detector (PAD) with a gold electrode (Dionex, ICS 3000, Sunnywale, Canada) using 200 mM NaOH as eluent. The Dionex CarboPac PA1 carbohydrate Analytical column (4 mm × 250 mm) was used for the analysis. The starch analysis was done on the extracted material by gelatinisation in an autoclave at 120°C for 90 min. The materials were then incubated with Na-acetate buffer containing amyloglucosidase and α-amylase for 16-h at 30°C. After incubation, the samples were centrifuged for 5 min at 13414 × *g*, the supernatant was collected and filtered through a 0.45 μm syringe filter before analysis. The starch concentration was determined by ion chromatography as glucose equivalent as described above.

### Antioxidative Enzymes

Approximately 200 mg of fresh leaf samples were collected at the end of the light period on day 12 (EL, 12:30 CEST) and immediately frozen in liquid nitrogen and stored at -80°C until analysis. The crude leaf extract for superoxide dismutase (SOD, EC 1.15.1.1), CAT (EC 1.11.1.6), and APX (EC 1.11.1.11) were assayed using the method of Elavarthi and Martin (2010). For SOD, 2 mL assay reaction mixture contained 50 mM phosphate buffer (pH 7.8) containing 2 mM EDTA, 9.9 mM L-methionine, 55 μM NBT, and 0.025% Triton-X100. Forty microliters of diluted (2×) sample and 20 μL of 1 mM riboflavin were added and the reaction was initiated by illuminating the samples under a 15 W fluorescent tube (Giannopolitis and Ries, 1977). During the 10-min exposure, the test tubes were placed in a box lined with aluminum foil. The box with the test tubes was placed on a slowly oscillating platform ∼12 cm from the light source. Duplicate tubes with the same reaction mixture were kept in the dark and used as blanks. Absorbance of the samples was measured immediately after the reaction was stopped at 560 nm. CAT activity was determined according to Aebi and Lester (1984) and APX activity was assayed using the method of Nakano and Asada (1981).

### Harvest

After 12 days, four plants from each species in each treatment were randomly selected for harvest. The total leaf area per plant was measured by a leaf area meter (LI-3100, Li-COR Inc., Lincoln, NE, USA). Then leaves and stems were separated, fresh weight was determined before the material was oven dried at 70°C for 24-h to determine leaf and stem dry weight (DW).

### Statistical Analysis

The statistical software R^2^ was used to evaluate the variation of parameters among the CL treatments. Data analysis was done using analysis of variance (ANOVA) and *P* < 0.05 was considered as significant. Homogeneity of variance was tested with Bartlett test and only the SOD data of *S. lycopersicum* were transformed using square root transformation of data, as the variance was not homogenous. The multiple comparisons of treatment means for each day of measurement were done considering the factor treatment by Tukey and Waller–Duncan k ratio *t*-test.

## Results

**FIGURE 1:**
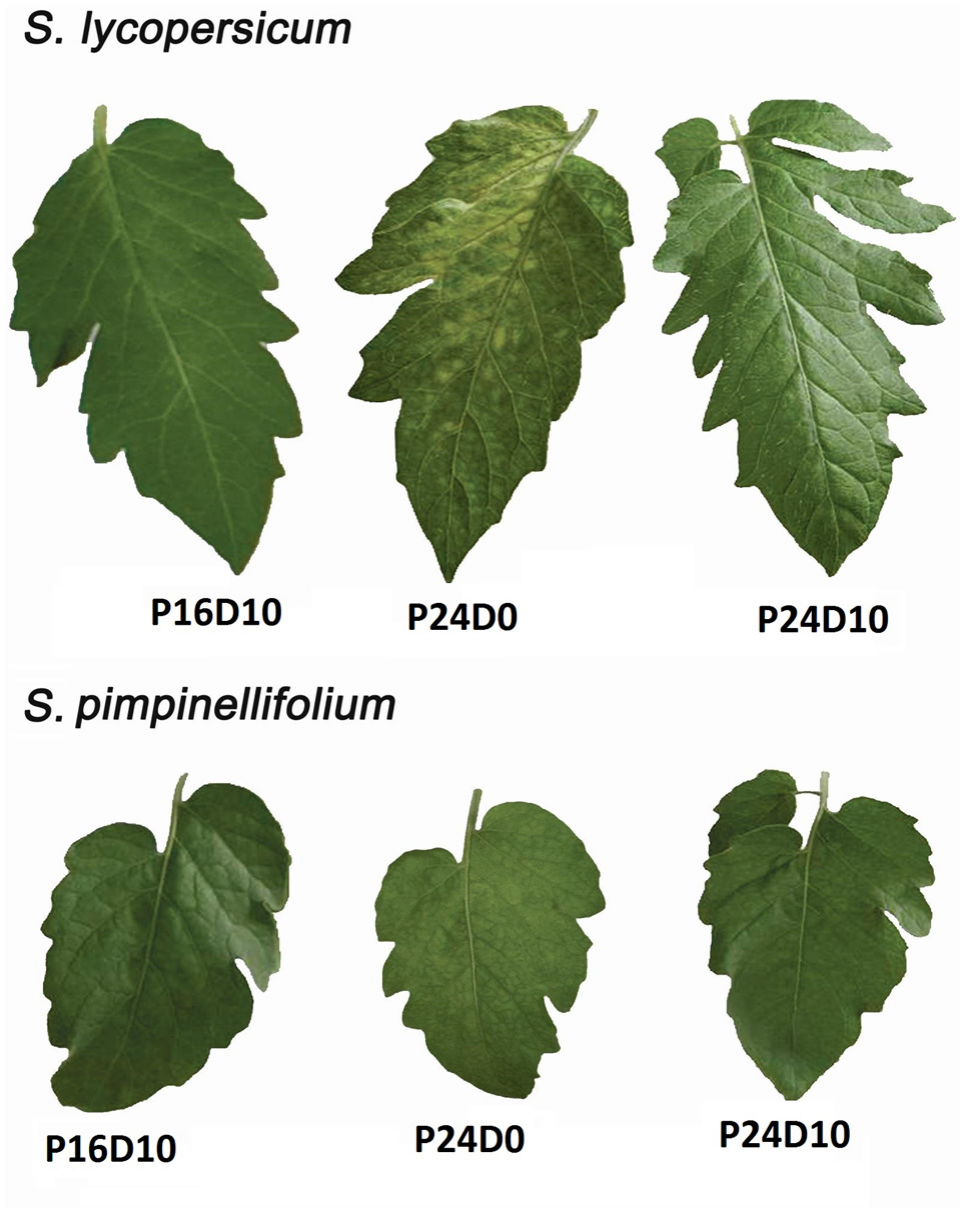
**Leaflets of *Solanum lycopersicum* and *S. pimpinellifolium* grown in control (P16D10), continuous light with constant temperature (P24D0) and continuous light with variable temperature (P24D10) conditions.** The photographs were taken after 12 days of start of CL exposure.

The development of chlorotic areas on the leaves became visible in *S. lycopersicum* after 8 days of CL, but only in the treatment with constant temperature (P24D0; **Figure 1**). No symptoms of leaf chlorosis were seen in *S. pimpinellifolium* in any of the three treatments. The development of the chlorotic areas in *S. lycopersicum* started from the base of the leaflets on leaf four and expanded toward the apex (**Figure 1**). Chlorotic areas were also seen on the top leaves above leaf four.

### Chlorophyll Fluorescence and Photosynthesis

**FIGURE 2:**
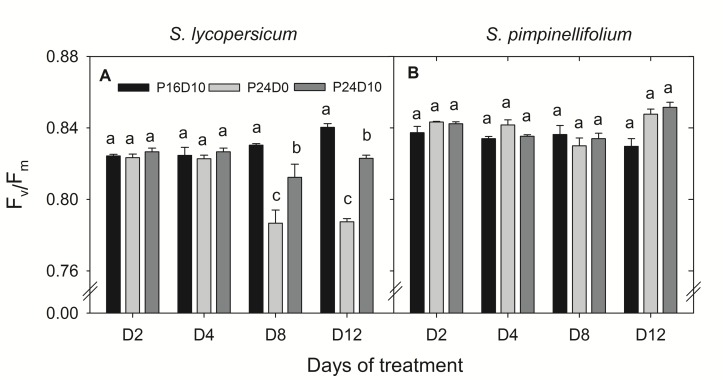
**Response of F_v_/F_m_ in *S. lycopersicum***(A)** and *S. pimpinellifolium***(B)** at different days (D) under P16D10, P24D0, and P24D10 growth conditions.** The F_v_/F_m_ data were taken at 21:30 CEST. Vertical bars are SEM (*n* = 3). Treatment means with different letters within each day are significantly different. The statistical analysis was done by the Tukey and Waller–Duncan k-ratio *t*-test.

The F_v_/F_m_ of *S. lycopersicum* was significantly lower on day 8 and 12 in plants from the CL treatments compared to P16D10 (**Figure 2A**). The values were lower in P24D0 and intermediate in P24D10. In contrast, there was no significant effect on the F_v_/F_m_ values of *S. pimpinellifolium* (**Figure 2B**). The P_N_ of CL-grown plants of *S. lycopersicum* were similar in the two photoperiods (**Table 2**). The diurnal variations of P_N_ (between 9:00 and 15:00) in both species were not significantly different at any of the 4 days in P24D0 treatment. In P24D10, the P_N_ at dark period (15:00) was significantly higher than at light period (9:00) on day 3, 6, and 9 for *S. lycopersicum*, but the diurnal variations were not statistically significant for *S. pimpinellifolium* (**Table 2**). In the period corresponding to the dark period, the P_N_ of *S. lycopersicum* was significantly higher in the P24D10 compared to the P24D0 conditions in two out of 4 days (**Table 2**). In *S. lycopersicum*, the P_N_ significantly increased in the P16D10 plants from day 3 to day 12 (*P <* 0.01), whereas the P_N_ in CL-grown plants were not significantly increased from day 3 to day 12 (*P* > 0.05). During the light period, the P_N_ of *S. pimpinellifolium* grown in P24D0 was only significantly reduced in comparison to the P16D10 on day 12 and the P_N_ in all the treatments of this species increased significantly (*P* < 0.05) during the experiment (**Table 2**). The daily carbon gain in both species was significantly higher in the CL treatments compared to P16D10 treatment, while it was significantly higher in P24D0 than P24D10 on days 6 and 9 in *S. pimpinellifolium* (**Table 3**).

**Table 2:** The rate of leaf photosynthesis (P_N_, μmol CO_2_ m^-2^ s^-1^) in *Solanum lycopersicum* and *S. pimpinellifolium* grown in three photoperiod treatments (P16D10, P24D0, and P24D10) where *P* gives the hours of light and D gives the difference in temperature between light and dark.

Day and time	*S. lycopersicum*			*S. pimpinellifolium*		
	P16D10	P24D0	P24D10	P16D10	P24D0	P24D10
3L 09:00	6.2 ± 0.2 a	5.3 ± 0.1 bA	5.0 ± 0.1 bB	4.5 ± 0.1 a	4.8 ± 0.3 aA	4.4 ± 0.4 aA
3D 15:00	-1.1 ± 0.1 c	4.9 ± 0.1 bA	6.0 ± 0.2 aA	-0.7 ± 0.1 b	5.0 ± 0.3 aA	4.4 ± 0.4 aA
6L 09:00	6.4 ± 0.3 a	5.9 ± 0.4 aA	5.3 ± 0.2 aB	5.9 ± 0.2 a	6.2 ± 0.2 aA	4.7 ± 0.1 bA
6D 15:00	-0.9 ± 0.1 b	6.0 ± 0.2 aA	6.9 ± 0.2 aA	-0.5 ± 0.1 b	6.2 ± 0.3 aA	5.3 ± 0.4 aA
9L 09:00	6.5 ± 0.2 a	5.0 ± 0.4 bA	4.7 ± 0.2 bB	6.4 ± 0.2 a	6.5 ± 0.9 aA	5.5 ± 0.4 bA
9D 15:00	-0.7 ± 0.1 c	4.7 ± 0.3 bA	6.4 ± 0.3 aA	-0.5 ± 0.1 c	6.7 ± 0.2 aA	5.4 ± 0.1 bA
12L 09:00	7.6 ± 0.1 a	5.4 ± 0.9 bA	5.5 ± 0.3 bA	7.7 ± 0.2 a	6.4 ± 0.2 bA	6.0 ± 0.1 bA
12D 15:00	-0.5 ± 0.1 b	5.4 ± 0.1 aA	5.8 ± 0.2 aA	-0.7 ± 0.1 b	6.2 ± 0.1 aA	6.2 ± 0.1 aA

*The P_*N*_ measurements of each day were taken at 09:00 (L) and 15:00 (D) at a PPFD of 150 μmol m^-*2*^ s^-*1*^ for the P24D0 and P24D10 plants during the light period. In P16D10 plants, P_*N*_ measured at a PPFD of 225 and 0 μmol m^-*2*^ s^-*1*^ for the light (L) and dark period (D), respectively. Treatment means with different letters (lowercase for each time of measurement and uppercase for each day of measurement) are significantly different and the statistical analysis was done by the Tukey and Waller–Duncan k-ratio *t*-test. Values represent mean ± SEM (*n* = 3).*

**Table 3:** The daily carbon gain (mmol CO_2_ m^-2^ d^-1^) in two species of tomato grown in three photoperiodic treatments.

Day	*S. lycopersicum*			*S. pimpinellifolium*		
	P16D10	P24D0	P24D10	P16D10	P24D0	P24D10
3	328 ± 11 b	445 ± 9 a	458 ± 7 a	240 ± 6 b	420 ± 24 a	382 ± 32 a
6	340 ± 18 b	514 ± 22 a	505 ± 14 a	324 ± 12 c	538 ± 8 a	424 ± 5 b
9	354 ± 14 b	425 ± 29 a	453 ± 21 a	356 ± 10 c	564 ± 13 a	470 ± 5 b
12	420 ± 9 b	464 ± 9 a	484 ± 20 a	428 ± 10 b	547 ± 12 a	527 ± 6 a

*The calculation was based on photosynthetic measurements on three plants on each of the days. Values represent mean ± SEM (*n* = 3).*

### Pigments

**FIGURE 3:**
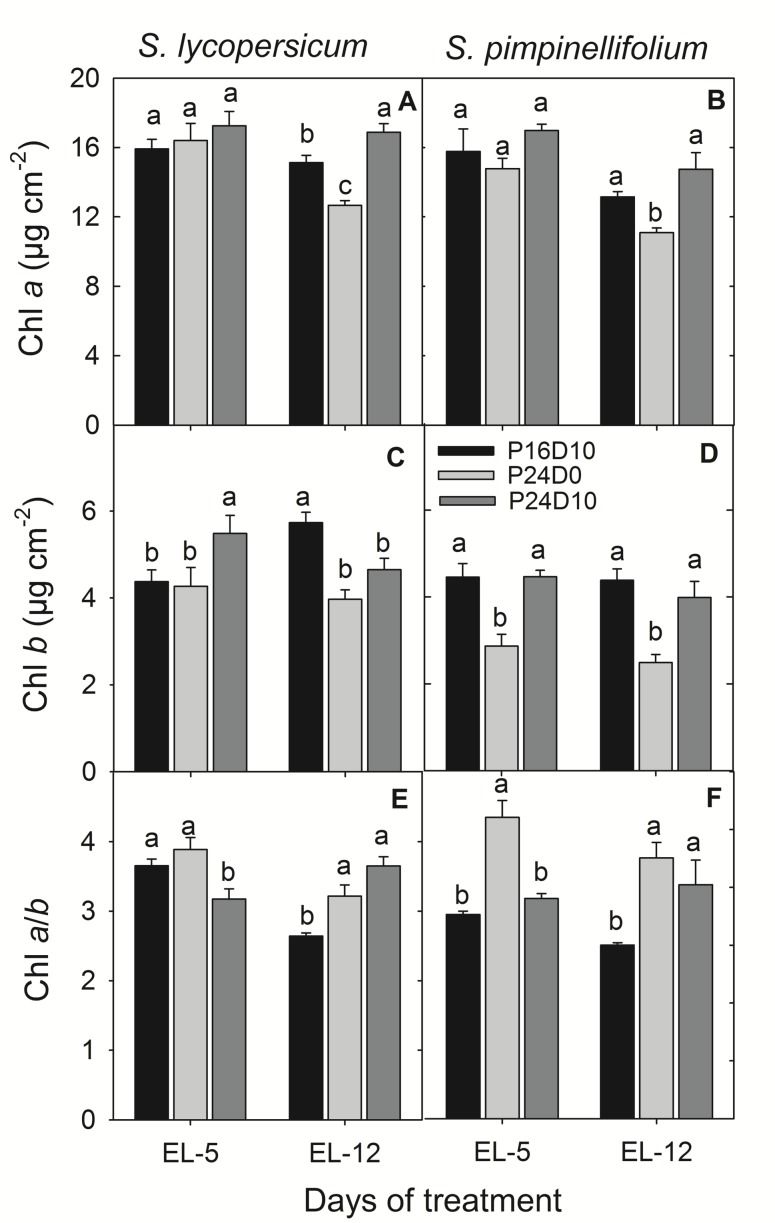
**Chl *a*, Chl *b* and Chl *a*/*b* ratio of *S. lycopersicum***(A,C,E)** and *S. pimpinellifolium***(B,D,F)** at different days under P16D10, P24D0, and P24D10 growth conditions.** The pigment data was taken at the end of the light period (EL, 12:30 CEST) of each day. Vertical bars are SEM (*n* = 4). Treatment means with different letters within each day are significantly different and the statistical analysis was done by the Tukey and Waller–Duncan k-ratio *t*-test.

There was no significant effect of CL on the leaf Chl *a* content in any of the species on day 5. After 12 days, the leaf Chl *a* content was significantly reduced in both species in P24D0. In *S. lycopersicum* leaf Chl *a* significantly increased in P24D10, while was unaffected in *S. pimpinellifolium* (**Figures 3A,B**). In contrast, the Chl *b* content in *S. lycopersicum* leaves was significantly higher in P24D10 than in P24D0 and P16D10 on day 5, whereas on day 12, CL-grown plants had significantly lower leaf Chl *b* contents than the P16D10 plants (**Figure 3C**). In *S. pimpinellifolium*, the leaf Chl *b* content was significantly lower in P24D0 than in the P16D10 and P24D10 on both days (**Figure 3D**). The leaf Chl *a*/*b* ratio was significantly higher in CL-grown plants of both species after 12 days (**Figures 3E,F**). The leaf Chl *a*/*b* ratio in P24D0 grown plants was higher in *S. pimpinellifolium* than in *S. lycopersicum* (**Figures 3E,F**).

### Carbohydrates

**FIGURE 4:**
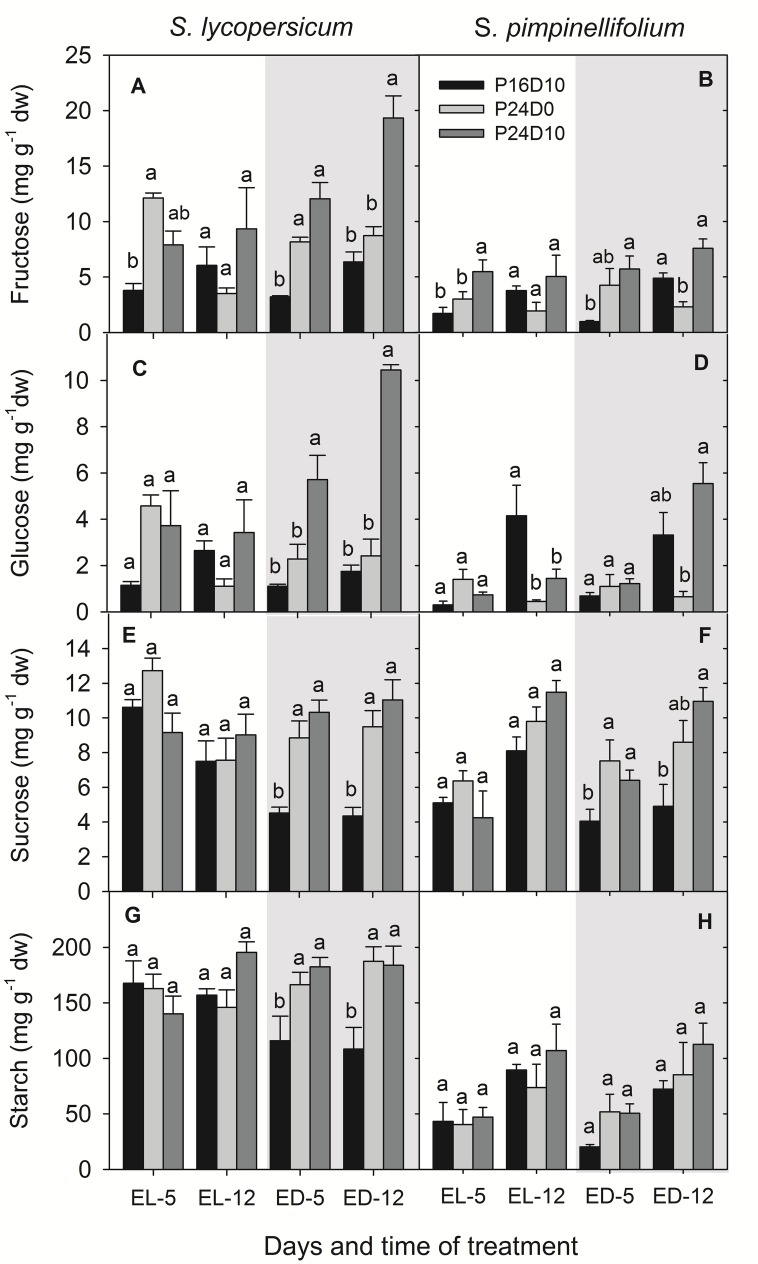
**Fructose **(A,B)**, glucose **(C,D)**, sucrose **(E,F),** and starch **(G,H)** contents in *S. lycopersicum* and *S. pimpinellifolium* leaves at different times of the day in P16D10, P24D0, and P24D10 growth conditions.** The data were collected at the end of light (EL, 12:30; white shadow) and at the end of dark (ED, 20:30; gray shadow) of each day. Vertical bars are SEM (*n* = 4). Treatment means with different letters within each time of measurement are significantly different and the statistical analysis was done by the Tukey and Waller–Duncan k-ratio *t*-test.

The content of sugars and starch in leaves of *S. lycopersicum* was significantly affected by CL (**Figure 4**) showing an accumulation of large amount of glucose and fructose especially, when the temperature was low during the period corresponding to the dark period in the P16D10 treatment (**Figures 4A,C**). At the end of the light period of P16D10 on day 5, the leaf glucose and fructose content in *S. lycopersicum* were higher in the CL treatments compared to the P16D10 treatment, whereas fructose and glucose contents at the end of the light period on day 12 were not significantly different (**Figures 4A,C**). At the end of the dark period (the dark period of P16D10), the glucose and fructose contents increased in the *S. lycopersicum* plants in P24D10.

In general, the fructose and glucose contents were higher in *S. lycopersicum* that in *S. pimpinellifolium* and a significant increase were seen between treatments (**Figures 4A–D**). There was no significant effect of treatment in any of the species on the leaf sucrose content at the end of the light period, though the values for sucrose content was significantly (*P* < 0.01) higher for *S. pimpinellifolium* on day 12 compared to day 5 (**Figures 4E,F**). In *S. lycopersicum*, the leaf sucrose content was significantly lower in the P16D10 plants at the end of dark period than in plants grown in both CL treatments while no significant differences were found between the plants grown in P24D0 and P24D10 at the end of dark period (**Figure 4E**).

The leaf starch content in *S. pimpinellifolium* was significantly (*P* < 0.001) lower than in *S. lycopersicum* (**Figures 4G,H**). Overall, the leaf starch contents did not significantly differ among the treatments by the end of the period corresponding to the light period in the P16D10 in any of the species, while both CL treatments lead to a higher accumulation of starch in *S. lycopersicum* by the end of what corresponds to the dark period in P16D10. In *S. pimpinellifolium* the starch content was not affected.

### Antioxidative Enzymes

**FIGURE 5:**
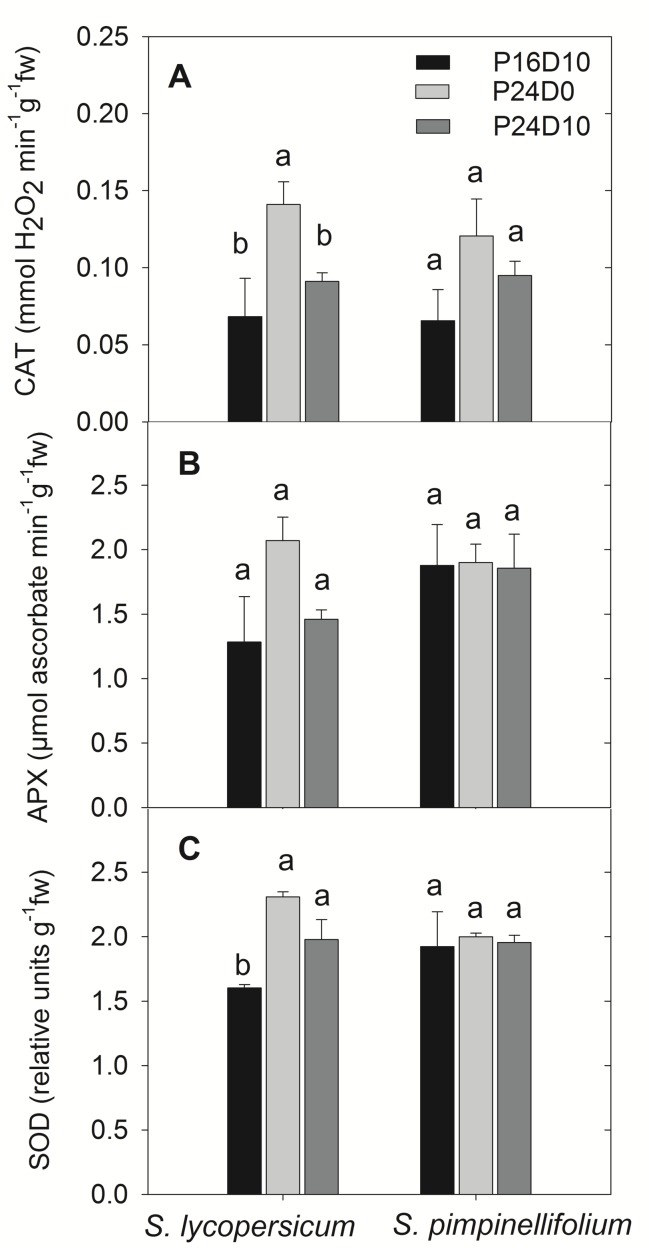
**Catalase (CAT, **A**), Ascorbate peroxidase (APX, **B**), and Superoxide dismutase (SOD, **C**) enzymes activities in the plants of *S. lycopersicum* and *S. pimpinellifolium* at different days in P16D10, P24D0, and P24D10 growth conditions.** The data were taken at the end of the light period on day 12 (12:30 CEST). Vertical bars are SEM (*n* = 4). Treatment means with different letters within each species are significantly different and the statistical analysis was done by the Tukey and Waller–Duncan k-ratio *t*-test.

The activities of antioxidative enzymes in leaves are shown for the end of light period. At the end of the light period on day 12, the activity of CAT were significantly higher in *S. lycopersicum* grown in P24D0 compared to P24D10 and P16D10, whereas no significant difference in CAT activity were seen in *S. pimpinellifolium* (**Figure 5A**). The APX activities in both species did not differ significantly (**Figure 5B**). In *S. lycopersicum*, the SOD activities were significantly higher in P24D0 and P24D10 conditions in comparison to the SOD activity in P16D10 plants, while no significant differences in SOD activities was seen in *S. pimpinellifolium* (**Figure 5C**).

### Harvest

**FIGURE 6:**
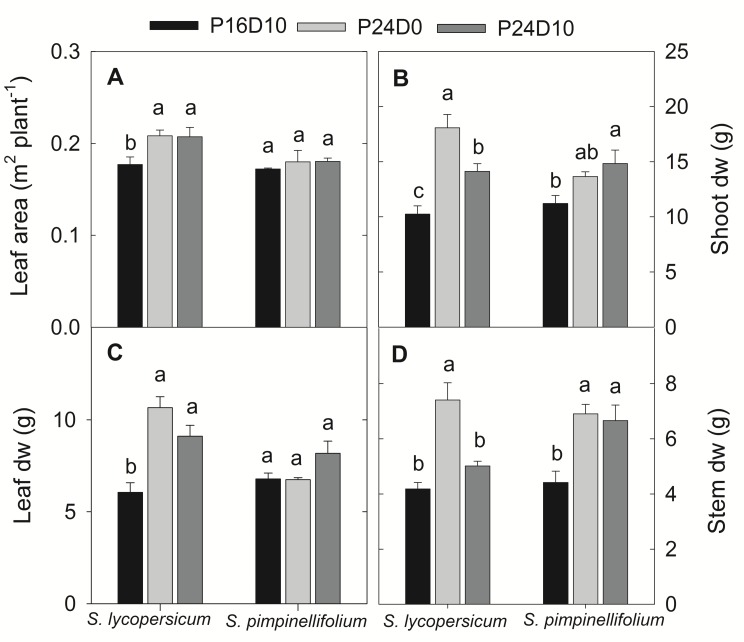
**Total leaf area **(A)**, shoot dry weight **(B)**, leaf dry weight **(C)**, and stem dry weight **(D)** of plants grown in respective growth conditions.** The harvests were done after 12 days of CL treatment. Vertical bars are SEM (*n* = 4). Treatment means with different letters within each species are significantly different and the statistical analysis was done by the Tukey and Waller–Duncan k-ratio *t*-test.

The total leaf area of *S. lycopersicum* increased significantly in CL, while no significant changes in total leaf area was observed in *S. pimpinellifolium* irrespective of treatments (**Figure 6A**). In both species, the CL-grown plants had highest shoot DW (**Figure 6B**). In *S. lycopersicum*, both leaf and stem DW were significantly higher in P24D0 followed by P24D10 and P16D10. In *S. pimpinellifolium*, there was no significant treatment difference in leaf DW but a significant increase in stem DW was seen in the CL compared to P16D10 (**Figures 6C,D**).

## Discussion

### Photosynthetic Reactions, Growth, and Antioxidants

The significant reductions in P_N_ in CL-grown *S. lycopersicum* in comparison to control during the light period at different days were primarily due to the measurement of P_N_ at lower PPFD. In *S. lycopersicum*, the P_N_ in the control plants increased significantly from day 3 to day 12, whereas no changes in P_N_ in CL-grown plants in time indicate that P_N_ might be affected by CL but it is less clear. In contrast, an increasing trend in P_N_ with time was seen in CL-grown *S. pimpinellifolium* plants except on day 12 indicating that the wild species was more efficient in assimilating CO_2_ under CL than *S. lycopersicum*. The effect of P_N_ in *S. lycopersicum* under P24D0 may be associated with the lower leaf Chl content and the reduction of F_v_/F_m_. The P_N_ was not limited by stomatal closure as the stomatal conductance was not affected, even increased in P24D0 in comparison to control and P24D10 (data not shown). The P_N_ decreased 14% at 200 μmol m^-2^ s^-1^ PPFD, when photoperiod increased from 12 to 24-h in *S. lycopersicum* after 2 weeks of CL and after 4 weeks, the P_N_ values were 7.6 and 4.4 μmol CO_2_ m^-2^ s^-1^ in 12 and 24-h photoperiodic treatments, respectively showing an increasing trend in P_N_ in control plants and a decreasing trend in 24-h photoperiodic plants (Matsuda et al., 2014). The continuous CO_2_ uptake and an increase in the total leaf area in *S. lycopersicum* under both CL conditions led to the increase in both leaf, stem, and shoot DW. These results are consistent with earlier works showing a higher dry matter accumulation in CL grown plants with the same DLI as in a control treatment (Ohyama et al., 2005a; Matsuda et al., 2014). The total DW of tomato seedling (*S. lycopersicum*) 60% increased when grown in CL with constant temperature (22°C) in comparison to CL with a variable temperature (28/16°C) maintaining the same mean daily temperature (Ohyama et al., 2005b). The shoot DW in P24D0 plants was 28% higher in comparison to plants grown in P24D10 for *S. lycopersicum*. The increased leaf area in our study under CL supports the findings of Ohyama et al. (2005a) but contradicts with other studies (Demers et al., 1998; Matsuda et al., 2014). The higher RH in P24D0 could influence the increase in leaf expansion as leaf expansion rate or leaf area increased with the increase of RH (Waldron and Terry, 1987; Hovenden et al., 2012). The increased leaf area induced by higher RH could have increased the total carbon gain by intercepting more light in the plants grown in CL treatments than in control plants and might have led to the increase in DW. The assimilate partitioning differed in the two plant species showing that the increase in shoot DW in CL-conditions was pronounced for both leaves and stems of *S. lycopersicum*, whereas mainly stem DW increased in *S. pimpinellifolium*. The *S. pimpinellifolium* could be more efficient in exporting assimilates than the *S. lycopersicum*. Furthermore, the positive effect of CL on shoot DW of *S. lycopersicum* was lower when the plants were exposed to temperature variation showing that excess carbohydrates may have been allocated to the roots.

The CL with constant temperature reduced the Chl *a* and *b* content in both species, which might be an effect of the extended photoperiod. Dorais (1992) reported that leaf Chl content in tomato and sweet pepper were negatively correlated with extension of the photoperiod. Globig et al. (1997) found that the Chl content increased with exposure to CL but decreased 40%, when chlorotic mottling became very apparent. We also saw a significant reduction in the leaf Chl content in *S. lycopericum* after 12 days in CL conditions accompanied by chlorotic areas on the leaves, supporting the suggestion that chlorotic development are linked to a decrease in Chl content. Furthermore, the leaf chlorotic development was accompanied with reductions in F_v_/F_m_ values after 8 days, but only in *S. lycopersicum*. In *S. pimpinellifolium* significant reductions of leaf Chl content did not result in lower F_v_/F_m_ nor any visible leaf chlorosis. An increased activity of CAT and SOD in *S. lycopersicum* grown in P24D0 suggested an increased production of ROS had taken place in this treatment. The generation of ROS in CL might damage the ultrastructure and function of chloroplasts, affect PSII activity and photosynthetic pigments leading to leaf chlorosis (Asada, 1994; Foyer et al., 1994). The reduction in F_v_/F_m_ in *S. lycopersicum* in P24D0 indicated the photodamage of PSII under CL. Moreover, the CL-induced ROS production may inhibit the repair of PSII by suppressing primarily the synthesis of *de novo* proteins (Murata et al., 2007). However, when *S. lycopersicum* was subjected to a period of lower temperature the CAT and APX enzyme activities were reduced in comparison to P24D0 showing less photooxidative pressure in P24D10 plants. The increased ROS scavenging enzyme activity may reduce CL-induced injury in some species as shown in pepper plants, but not in eggplant (Murage and Masuda, 1997). The lack of increase in antioxidative enzyme activities in the leaves of *S. pimpinellifolium* showed that the absorbed photon energy was utilized for CO_2_ assimilation thus no enhanced generation of ROS due to absence of excess energy thus showing no photooxidative damage and photosynthetic reduction. Moreover, a higher Chl *a*/*b* ratio indicated that the *S. pimpinellifolium* was more efficient in acclimating to CL possibly by reducing the light-harvesting chlorophyll–protein complexes (Leong and Anderson, 1984). Velez-Ramirez et al. (2014) suggested that the formation and transition of the light-harvesting antenna complexes changes in tomato under CL and these changes were related to a lower expression of the *CAB-13* gene responsible for the type III light-harvesting Chl *a*/*b* binding protein in CL-sensitive plants in comparison to the CL-tolerant plants. They also found that the wild tomato species *S. pimpinellifolium* is CL-tolerant or CL-sensitive depending on the accession. However, the accession number of *S. pimpinellifolium* in this study was unknown and this wild tomato species performed well under CL indicating insensitive to CL (source^3^). Therefore, we might use the CL-tolerant *S. pimpinellifolium* accession in this study and not the one (*S. pimpinellifolium* LA1589) that has been reported as CL-sensitive by Velez-Ramirez et al. (2014).

### Carbohydrates

The higher daily carbon gain in CL-grown plants was not directly reflected in a higher leaf carbohydrate content. The increase in leaf sucrose and starch contents in *S. lycopersicum* by the end of the period corresponding the light period was similar for plants grown in all three treatments. On the other hand, the content of both sucrose and starch was reduced in the control treatment by the end of the dark period compared to the CL-grown plants. This reduction in carbohydrates during the dark is caused by dark-induced turnover of sucrose and starch, which does not occur in CL where the starch degrading enzymes are inhibited or down-regulated (Zeeman et al., 2007). Instead a continuous supply of assimilates support the carbohydrate pool in the CL-grown plants, as shown by the increase in hexoses (fructose and glucose) in *S. lycopersicum* exposed to a diurnal variation in temperature. This increase of soluble sugars could be due to a decreased respiration rate and a limited sucrose export at low temperature (Ap Rees et al., 1988). Low temperature increases the SPS and invertase enzyme activities leading to sucrose and hexose accumulation in spinach and potato (Richardson et al., 1990; Guy et al., 1992). Tomato plants grown under fluctuating diurnal temperature had total non-structural carbohydrate levels on average 8 mg g^-1^ greater than plants grown under a more constant temperature (Gent, 1984) which was 18 mg g^-1^ compared to our data (mean values of day 12). As the CL conditions were associated with a higher carbon gain and a higher shoot DW, it is most likely that the extra assimilates were directly invested in new biomass of both shoot and roots. In the past, several studies have reported an increased leaf starch and sugar contents of plants grown in CL (Bradley and Janes, 1985; Dorais et al., 1996; Demers and Gosselin, 2002) and they have suggested that the increased carbohydrate accumulation was responsible for down-regulation of P_N_ and development of leaf chlorosis. A strong short term temperature drop alleviated the negative effects of CL, but did not affect the growth (Ikkonen et al., 2015) and it was suggested that it was due to a reset of the diurnal rhythms. However, in these studies the maintenance of similar light intensities across treatments resulted in treatment differences in DLI. We measured a slight decrease in P_N_ over time in CL conditions, but as the carbohydrate contents were not severely increased, a direct relation between P_N_ and carbohydrate accumulation does not seem valid. Instead, it can be suggested that the altered carbohydrate metabolism, in terms of starch homeostasis, causing all assimilates to be directly exported from the chloroplast and into sucrose metabolism could possibly lead to a decreasing cellular instability causing reductions in P_N_ over time. In potato, the leaf starch concentration was higher in plants grown in a 18/6-h light/dark treatment than in a 24-h CL treatment at the end of the 18-h light period, while at the end of 6-h dark the values remained the same, due to impaired starch degradation in light (Stutte et al., 1996) which was also shown in cucumber (Wolf and Langerud, 2006). These studies in combination with our study demonstrated that an increase in starch accumulation in CL grown plants is merely explained by the absence of diurnal variation in starch contents under CL conditions. Maintaining the same DLI and considering the time of the day for measuring of carbohydrates is crucial, when studying the effects of CL on leaf carbohydrate metabolism and photosynthesis.

Soluble sugars like sucrose, glucose, and fructose might act as ROS scavenger when they are present in higher concentration by reducing oxidative phosphate pathway (OPP) and by increasing the biosynthesis of carotenoid and ascorbate compounds (Van den Ende and Valluru, 2009). The hyper-accumulation of glucose and fructose in P24D10 leaves may also play as potential ROS scavenger to avert the CL-induced photodamage. The ROS production and the relationship between ROS and antioxidants together with the role of sugars as ROS scavenger should be further investigated to improve the understanding of the photodamage of leaves subjected to CL.

## Conclusion

Both *S. lycopersicum* and *S. pimpinellifolium* grown in CL showed an increase in the carbon gain and DW due to the continuous P_N_ reflecting continuous carbon fixation. The wild species was not affected by CL with respect to the F_v_/F_m_, P_N_ and did not develop chlorotic areas and no increase in antioxidative enzymes. However, the daily variation in temperature (P24D10) alleviated the effect of CL in the cultivated species – no symptoms of leaf chlorosis, no decrease in leaf Chl content, no increase in CAT and APX enzyme activities and a significantly higher F_v_/F_m_ than P24D0. The effect of CL is suggested to be related to an increased light utilization efficiency resulting in higher CO_2_ fixation at lower quantum flux, expanded leaf area and altered carbohydrate metabolism directing the assimilates directly into dry matter production continuously. However, the expanded leaf area possibly induced by higher RH in CLCT treatment might have resulted in higher carbon gain through enhanced light interception. The study shows that the DLI and time of harvest of metabolites should be taken into account before conclusions are drawn in studies with different photoperiodic treatments, but a lower temperature during the “night” of CL treatments might alleviate the negative effect of CL and increase the biomass. Since the wild species is insensitive to CL stress, breeders should include wild species when selecting cultivars for extended or continuous lighting.

## Conflict of Interest Statement

This work was a part of a Ph.D. project supported by a grant from Aarhus University and the project ERDF GreenGrowing, NSR region of the European Union and Dynalight and SmartGrid funded by the Ministry of Food, Agriculture and Fisheries.

## Footnotes

1 http://www.rareplants.de

2 http://R-project.org/

3 http://www.rareplants.de
